# Induced bacterial sickness causes inflammation but not blood oxidative stress in Egyptian fruit bats (*Rousettus aegyptiacus*)

**DOI:** 10.1093/conphys/coac028

**Published:** 2022-04-25

**Authors:** David Costantini, Maya Weinberg, Lilla Jordán, Kelsey R Moreno, Yossi Yovel, Gábor Á Czirják

**Affiliations:** Unité Physiologie moléculaire et adaptation (PhyMA), UMR 7221, Muséum National d’Histoire Naturelle, CNRS, CP32, 57 rue Cuvier, 75005 Paris, France; Department of Zoology, Tel Aviv University, 6997801 Tel Aviv, Israel; Department of Wildlife Diseases, Leibniz Institute for Zoo and Wildlife Research, Alfred-Kowalke-Str. 17, 10315 Berlin, Germany; Behavioural Ecology Group, Department of Systematic Zoology and Ecology, ELTE Eötvös Loránd University, Pázmány Péter sétány 1/C, 1117 Budapest, Hungary; Department of Zoology, Tel Aviv University, 6997801 Tel Aviv, Israel; Department of Zoology, Tel Aviv University, 6997801 Tel Aviv, Israel; Sagol School of Neuroscience, Tel Aviv University, 6997801 Tel Aviv, Israel; Department of Wildlife Diseases, Leibniz Institute for Zoo and Wildlife Research, Alfred-Kowalke-Str. 17, 10315 Berlin, Germany

**Keywords:** oxidative stress, innate immunity, inflammation, infection, extracellular pathogen, ecoimmunology, bats, antioxidant

## Abstract

Bats are particularly interesting vertebrates in their response to pathogens owing to extremes in terms of tolerance and resistance. Oxidation is often a by-product of processes involved in the acute phase response, which may result in antimicrobial or self-damaging effects. We measured the immunological and oxidative status responses of Egyptian fruit bats (*Rousettus aegyptiacus*) to a simulated bacterial infection using lipopolysaccharide injection. As expected, experimental bats exhibited increases in two humoral immunological markers. However, they surprisingly did not show any effects across two markers of oxidative damage and four antioxidant markers. We propose that this lack of effects on oxidative status may be due to a reduction in cell metabolism through sickness behaviours or given life history traits, such as a long lifespan and a frugivorous diet. Finally, the consistency in the pattern of elevation in haptoglobin and lysozyme between current and previous findings highlights their utility as diagnostic markers for extracellular infections in bats.

## Teaser

Relying on a simulated bacterial infection in Egyptian fruit bats, we identify haptoglobin and lysozyme as potential diagnostic markers for extracellular infections in bats and find no relationships between inflammation and oxidative stress.

## Introduction

Pathogens may be drivers of behavioural and life history diversification and even cause dramatic population collapses driving species to the border of extinction (e.g. Lips *et al.*, 2006; McCallum *et al.*, 2009). As a consequence, animal species are critically reliant on the efficacy of their immune system to protect themselves against pathogens, to mitigate their activity or to clear off pathogens from their bodies. Animals, however, are not all equipped with the same defence toolkits nor do they show the same degree of responsiveness to all kinds of pathogens.

Bats are particularly special with respect to their pathogen load because they are reservoirs of many important zoonotic pathogens without developing any ostensible symptoms (Baker *et al.*, 2013; Mandl *et al.*, 2018; Moreno Santillán *et al.*, 2021; Wibbelt *et al.*, 2010). Lack of clinical symptoms is particularly interesting in relation to a high number of viral strains and other intracellular pathogens they harbour, while, on the other hand, bats show standard-to-extreme pathology following infection with certain extracellular pathogens, such as bacteria and fungi (Brook and Dobson, 2015). An important question then is why bats show variation in tolerance or resistance to pathogens. One answer to this question might lie in the physiological costs associated with an immune response that would impinge on the expression of life-history traits, such as reproduction.

Alteration of the oxidative status homeostasis (e.g. increase in molecular oxidative damage and change in antioxidant levels) may be one important physiological effect of immune response (Costantini and Møller, 2009). Leukocytes contain a multicomponent enzyme complex, the nicotinamide adenine dinucleotide phosphate (NADPH) oxidase that is responsible for the production of reactive oxygen species during an immune response (Babior, 2004). This mechanism is particularly relevant during the acute phase response (APR), which is a systemic innate reaction to disturbances in homeostasis caused by infective agents. To this end, during an APR, leukocytes increase their oxygen uptake (the so-called respiratory or oxidative burst). Reactive oxygen species produced by immune cells have a cytotoxic potential against pathogens. However, they can also cause oxidative damage to important biomolecules like proteins, lipids or nucleic acids, potentially leading to pathological consequences for the organism, making APR one of the costliest immune responses (Lee, 2006).

By measuring immunological and physiological markers, we can better determine individual variation in tolerance or resistance to a given pathogen and, possibly, respond with the best conservation approaches to improve wildlife health (Ohmer *et al.*, 2021). The utility of physiological markers to detect some subclinical infections in bats can be even more important when assessing the health status because they often lack clinical signs of infection. Thus, markers might help to isolate individuals that could be sick and, possibly, to monitor the effects of pharmacological treatments on their health status.

In this study, we have induced an APR in Egyptian fruit bats (*Rousettus aegyptiacus*) and we have monitored changes in inflammatory and oxidative status markers over a period of 48 hours. To this end, we injected bats with lipopolysaccharide (LPS, an endotoxin of gram-negative bacteria cell walls used experimentally to simulate a bacterial infection) and compared values of multiple markers with those of control bats. If bats are physiologically tolerant to short-term inflammation, we expected that LPS bats would have similar levels of all markers of oxidative status to those of control bats.

## Materials and Methods

### Study area and experimental design

Twenty-six adult male bats were captured from a cave roosting colony in Herzliya, Israel, in June 2020. They were checked for the presence of ectoparasites and treated topically with selamectin (Stronghold 15-mg Spot-On Solution for Puppies and Kittens, Zoetis). They were housed together in the experimental room (245 cm × 200 cm × 210 cm) for 4 days for a general health check and acclimation to captivity housing and feeding. The temperature in the experimental room was maintained at 25°C using a central AC system. Natural dark–light cycle of 12-hour duration each was enabled by an automatic clock. A variety of diced fruit (banana, apple and melon) of 150 g per individual was provided daily. All individuals were marked with a unique symbol using hair bleach on their backs for identification purposes.

Individuals were assigned to the control group and treatment group aleatory until we had a balanced sample size. Individuals in the treatment group were injected subcutaneously with a solution of *Escherichia coli* O111:B4 LPS (Sigma Alrdich, L2630) diluted in sterile phosphate-buffered saline (PBS, Sigma-Aldrich, P5493) to a concentration of 2 mg/ml. Control animals received PBS only. Each animal was injected with a volume (range: 0.12–0.18 ml) adjusted to their body weight in order to have a same dose of LPS per kg of body weight (2 mg LPS/kg b.w.) for all individuals. Dosage was determined in a preliminary experiment comparing the clinical outcome (body temperature elevation, visible lethargy, and joint swelling) of injecting 2 or 4 mg LPS/kg b.w.

The data collection was conducted in two rounds. Each round 10 individuals were assigned for the treatment group, and 3 individuals were assigned as a control group. For both rounds, the bats were handled three times, consisting of three-time points for data collection (pre-injection, 24- and 48-hour post-injection). During handling, challenged and control bats were removed from the colony to measure body weights and collect blood samples. All individuals were offered mango juice immediately after the following handling. The rounds were each performed at the same times of the day to maintain physiological uniformity. For both rounds, approximately 1 ml of blood was obtained via venipuncture with micro-container separation gel tubes (BD SST Serum Tube with Separating Gel) from the antebrachial or the wing vein using different locations for each sampling. Blood was kept cold and later centrifuged at 10 000 RPM for 3 minutes for complete separation between the cells and the serum. Both the clot and the serum were collected to a new Eppendorf tube and stored at −80°C until further analysis. Samples were transported to Germany and later to France without interruption of the cold chain.

### Laboratory analyses

We measured multiple physiological metrics relying on established methods for bats (Costantini *et al.*, 2019; Fritze *et al.*, 2019, 2021; Moreno *et al.*, 2021; Voigt *et al.*, 2020). Specifically, we measured two immunological markers (haptoglobin and lysozyme in serum), two markers of oxidative damage (protein carbonyls in red blood cells and reactive oxygen metabolites [ROMs] in serum) and four antioxidant markers (superoxide dismutase, glutathione peroxidase [GPx] and total thiols in red blood cells; non-enzymatic antioxidant capacity in serum). Briefly, we measured (i) haptoglobin using the commercial kit PHASE Haptoglobin Assay (Tridelta, Ireland); (ii) lysozyme using the lysoplate assay, which was adapted to low sample volumes; (iii) protein carbonyls using the OxiSelect Protein Carbonyl ELISA Kit (Euromedex, France); (iv) ROMs using the d-ROMs assay (Diacron International, Italy); (v) GPx using the Ransel test (Randox Labs, France); (vi) superoxide dismutase using the Ransod test (Randox Labs, France); (vii) concentration of total thiols using the -SHp test (Diacron International, Italy); and (viii) the non-enzymatic antioxidant capacity using the OXYAdsorbent test (Diacron International, Italy).

## Statistical Analyses

We ran all statistical analyses using RStudio version 1.1.463 (R Core Team, 2020). We selected *P* < 0.05 as statistically significant. We performed linear mixed models (package lme4) to test the effects of LPS injection on inflammatory and oxidative status markers. In each model, we included, as fixed factors, treatment group (control vs. LPS), sampling day (pre-injection of LPS, 24- and 48-hour post-injection of LPS) and the interaction between treatment group and sampling day. We also included individual identity as a random factor to control for repeated sampling of individuals. We performed post-hoc comparisons using the Tukey test to explore significant interactions.

## Results

**Figure 1:**
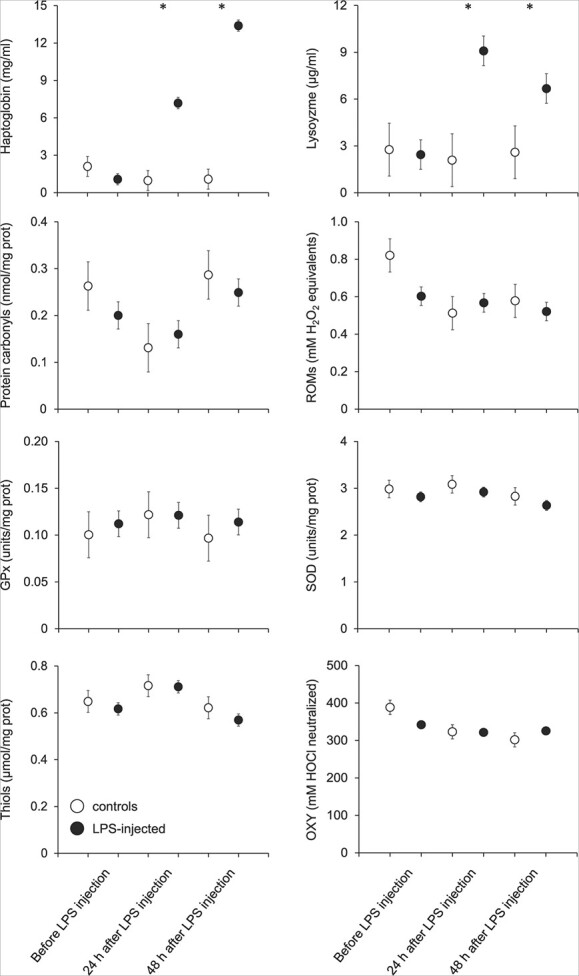
Effects of LPS injections on inflammatory and oxidative status markers in bats. ROMs, reactive oxygen metabolites; GPx, glutathione peroxidase; SOD, superoxide dismutase; OXY, non-enzymatic antioxidant capacity. The data are shown as least square means ± s.e. * indicates a significant difference between groups.

Control and LPS bats had similar values of all blood-based markers before the start of the experiment (Fig. 1). As compared to control bats and to pre-LPS injection values, we found that haptoglobin increased significantly over the course of the experiment in LPS-injected bats, with a peak at 48-hour post-injection (Table 1 and Fig. 1). Haptoglobin concentration increased by a factor of 6.7 and of 12.4 at 24- and 48-hour post-injection, respectively. We also found that, as compared to control bats and to pre-LPS injection values, lysozyme increased significantly after LPS injection, but, conversely to haptoglobin, it reached a peak 24-hour post-injection (Table 1 and Fig. 1). Lysozyme concentration increased by a factor of 3.7 and of 2.7 at 24- and 48-hour post-injection, respectively. By contrast to inflammatory markers, all oxidative status markers were not affected by the LPS injection (Table 1 and Fig. 1). Irrespective of the treatment group, protein carbonyls, thiols, ROMs and OXY varied over the duration of the experiment (Table 1).

**Table 1:** Outcomes of linear mixed models performed to quantify the effects of the LPS injection on inflammatory and oxidative status markers

**Variable**	**Factor**	**F**	***P***
Haptoglobin	Experimental group	50.29	< 0.001
	Sampling day	35.50	< 0.001
	Exp. group × sampling day	66.22	< 0.001
Lysozyme	Experimental group	11.01	0.003
	Sampling day	4.33	0.019
	Exp. group × sampling day	9.97	0.0003
Protein carbonyls	Experimental group	0.47	0.50
	Sampling day	4.78	0.013
	Exp. group × sampling day	0.71	0.49
ROMs	Experimental group	0.65	0.43
	Sampling day	6.05	0.005
	Exp. group × sampling day	2.52	0.092
GPx	Experimental group	0.01	0.91
	Sampling day	1.46	0.24
	Exp. group × sampling day	0.55	0.58
SOD	Experimental group	1.51	0.23
	Sampling day	1.95	0.15
	Exp. group × sampling day	0.01	0.99
Thiols	Experimental group	0.89	0.36
	Sampling day	5.38	0.008
	Exp. group × sampling day	0.21	0.82
OXY	Experimental group	0.33	0.57
	Sampling day	5.70	0.005
	Exp. group × sampling day	2.20	0.12

ROMs, reactive oxygen metabolites; GPx, glutathione peroxidase; SOD, superoxide dismutase; OXY, non-enzymatic antioxidant capacity

## Discussion

We found that the experimental induction of an APR by LPS injection caused an inflammatory status that lasted at least over the 48 hours of the experiment. By contrast to inflammatory markers, the LPS injection did not affect any of the six oxidative status markers (including damage, and both enzymatic and non-enzymatic antioxidants) analysed in this study.

Our results on inflammatory markers replicate previous findings on this species (using higher dosages; Moreno *et al.*, 2021) and are partly in agreement with prior work on other bat species. For example, injection of a similar dose of LPS in Nathusius’ pipistrelles (*Pipistrellus nathusii*) caused an increase of haptoglobin by a factor of 7.8 in migratory individuals, whereas it did not affect haptoglobin during the pre-migration period (Voigt *et al.*, 2020). Haptoglobin-associated APR is clearly influenced by the life-history stage of the bats during antigen exposure. Besides the effects of migratory phase (Voigt *et al.*, 2020), hibernating greater mouse-eared bats (*Myotis myotis*) challenged with the fungal antigen zymosan increased the levels of haptoglobin (Fritze *et al.*, 2019), while there was no effect during active phase in summer months (Seltmann *et al.*, submitted). The clinical importance of haptoglobin has been described during natural infections with *Pseudogymonascus destructans*, the etiologic agents causing white-nose syndrome, in both North American (Field *et al.*, 2015) and European bats (Fritze *et al.*, 2021). Based on these results, we conclude that haptoglobin is a major acute phase protein in bats, while lysozyme is an intermediate one, but we have much more limited information on this antibacterial enzyme in bats (Costantini *et al.*, 2019; He *et al.*, 2019; Moreno *et al.*, 2021).

Similarly to our results, injection of a similar dose of LPS in Nathusius’ pipistrelles did not affect ROMs nor OXY levels (Voigt *et al.*, 2020). Also, prior work did not find any effect of a zymosan challenge on oxidative status markers in hibernating greater mouse-eared bats (Fritze *et al.*, 2019). By contrast, LPS injection induced a significant increase of ROMs in short-tailed fruit bats (*Carollia perspicillata*) (Schneeberger *et al.*, 2013). Any effect of antigen dose and time of sampling can be ruled out in explaining the discrepancies between these studies because of their similar experimental designs. It might be that variation in life histories between species had a significant impact on their capacity to deal with oxidative stress (Brunet-Rossinni, 2004; Costantini, 2014). Prior work has shown that frugivorous bats have higher circulating non-enzymatic antioxidant capacity compared to animalivorous and omnivorous species (Schneeberger *et al.*, 2014). While both short-tailed and Egyptian fruit bats have a significant amount of fruit in their diet, recent studies showed that *Carollia* species also eat relatively large numbers of insects (York and Billings, 2009). The antioxidants originating from fruits, and that are stored in tissues like liver, might allow both species to cope with pro-oxidants generated during normal physiological processes. However, it might be that the amount of stored antioxidants is not enough to enable short-tailed fruit bats to mitigate the costs associated with an immune challenge. Another potential explanation for our results might lie with a lower ROS production by leukocytes or a different regulation of the oxidative burst in bats as compared to other taxa. For example, prior work found differences in phagocytic respiratory burst between bats and mice (Pikula *et al.*, 2020).

In a previous experiment, we found that Egyptian fruit bats injected with LPS develop classical illness symptoms, including fever, weight loss, anorexia and lethargy (Moreno *et al.*, 2021). In addition, they also isolated themselves from the group by leaving the social cluster and avoiding contact and ceased foraging outdoors for at least two nights (Moreno *et al.*, 2021). This strong reduction in activity might have determined a significant reduction of metabolic rate. Cell respiration is responsible for a large production of reactive oxygen species, thus bats could have buffered any increase in oxidative stress owing to the immune stimulation by reduction of metabolic activity needed to sustain other functions, such as foraging flights.

Bats are exceptionally long-lived mammals given their body size and metabolic rate (Wilkinson and South, 2002). It has been hypothesized that a reduced cellular production of some ROS (Brunet-Rossinni, 2004) and a divergent selection for genes that may repair molecular damages caused by free radicals (Foley *et al.*, 2018; Huang *et al.*, 2020; Jebb *et al.*, 2018) might explain this exceptional longevity. However, Wilkinson *et al.* (2021) suggested that the exceptional longevity of bats might also be due to an augmented immune response as compared to other taxa. If so, bats might have indeed evolved a capacity to develop a moderate ROS production by activated phagocytes, a question that would deserve special attention. This possibility has some support from the observation that the immune response in bats generates lower inflammation than that in other taxa, without the development of fever, leukopenia or pathological outcomes associated with infection (e.g. Baker *et al.*, 2013; Mandl *et al.*, 2018; Pavlovich *et al.*, 2018; Xie *et al.*, 2018).

In conclusion, the consistency between current and previous findings in the pattern of elevation of haptoglobin and lysozyme following an immune stimulation (Moreno *et al.*, 2021) highlights their utility as diagnostic markers. Together, they can provide valuable information on the magnitude and timing of an APR in an individual who is suspected to have an extracellular infection. In order to get insight into the mechanisms underlying the cross-talk between immune response and oxidative status, we suggest adding the direct measurement of neutrophil oxidative burst capacity in future studies as recently done in birds (Huber *et al.*, 2017). Finally, our work adds to a growing number of studies suggesting that bats have evolved mechanisms promoting homeostasis of the oxidative status during infections and preventing oxidative stress and the potential detrimental effects on fitness.

## Funding

LJ received an Erasmus+ scholarship from the Eötvös Loránd University, Budapest, Hungary. GÁC was supported by funds from the Leibniz Institute for Zoo and Wildlife Research, Berlin. KRM was supported by the Zuckerman STEM Leadership Program.

## Data Availability

The full dataset is provided as supplementary material.
